# 

**Published:** 1896-06-13

**Authors:** 


					TBk HOBPITAL. ABSOLUTELY PURE, therefore BEST, J?E 13th. 1896-
CADBURY'S ww ^ eAOBURY'S.
cocoa ?H| Ju^ cocoa \veh/,r !
f~\ - " The standard of highest purity at present NO ALKALIES USED,
attainable."?Lancet,
WICH H0SP*.7Al
No. 507.^vol. XX. WITH EXTRA NURSING SUPPLEMENT Satubday,
June 13tji, 1896.
SPECIAL HOSPITAL SUNDAY NUMBER.
WITH ILLUSTRATED SUPPLEMENT.

				

